# Application of iTRAQ Reagents to Relatively Quantify the Reversible Redox State of Cysteine Residues

**DOI:** 10.1155/2012/514847

**Published:** 2012-07-15

**Authors:** Brian McDonagh, Pablo Martínez-Acedo, Jesús Vázquez, C. Alicia Padilla, David Sheehan, José Antonio Bárcena

**Affiliations:** ^1^Department of Biochemistry and Molecular Biology, University of Córdoba and IMIBIC, 14071 Córdoba, Spain; ^2^Cardiovascular Proteomics Laboratory, National Center for Cardiovascular Research, 28026 Madrid, Spain; ^3^Department of Biochemistry, University College Cork, Cork, Ireland

## Abstract

Cysteines are one of the most rarely used amino acids, but when conserved in proteins they often play critical roles in structure, function, or regulation. Reversible cysteine modifications allow for potential redox regulation of proteins. Traditional measurement of the relative absolute quantity of a protein between two samples is not always necessarily proportional to the activity of the protein. We propose application of iTRAQ reagents in combination with a previous thiol selection method to relatively quantify the redox state of cysteines both within and between samples in a single analysis. Our method allows for the identification of the proteins, identification of redox-sensitive cysteines within proteins, and quantification of the redox status of individual cysteine-containing peptides. As a proof of principle, we applied this technique to yeast alcohol dehydrogenase-1 exposed in vitro to H_2_O_2_ and also in vivo to the complex proteome of the Gram-negative bacterium *Bacillus subtilis*.

## 1. Introduction

The dynamic nature of the proteome ensures that the cell is able to respond to perturbation of environmental, genetic, biochemical, and pathological conditions. How the proteome responds to these stimuli is of considerable interest as it can relate to the cell's stress response and can take the form of posttranslational modifications and interprotein interactions with subsequent effects on translation and transcription. Improvements in mass spectrometry has led to the development of a number of techniques to quantify the relative protein abundance within a given sample. These include isotope-coded affinity tags (ICATs) [1], stable isotope labeling of amino acids in cell culture (SILAC) [2], and isobaric tags for relative and absolute quantification (iTRAQ) [3]. However, measuring the relative quantity of a protein between two samples does not tell us anything about the activity of the protein itself. This is especially important in reference to redox proteins that contain thiol switches susceptible to activation or inactivation.

Cysteine is the most important redox-responsive amino acid within proteins largely due to the wide range of oxidation states that sulfur can occupy—so called, “sulfur switches” [4]. Indeed, it has been demonstrated that cysteines are characterized by the most extreme conservation pattern, being highly conserved in functional positions of proteins but poorly conserved otherwise [5]. Within an individual protein there may be a number of cysteines which could allow for multiple thiol modifications. Cysteines often form part of active sites, allowing for the protein to be switched on or off depending on redox state. One of the best-known examples of this is glyceraldehyde 3-phosphate dehydrogenase [6]. In proteins where cysteine is not within the active site, activity can be modulated by changing conformation or by influencing its regulatory role, for example, iron sulfur complexes (ISCs) in aconitase possess cysteines required for its activity [7]. Interactions with other proteins or molecules are another feature of cysteines that can affect protein activity. Allosterically regulated proteins that require an activator are sometimes based on a thiol exchange interaction involving cysteines, for example, pyruvate kinase uses fructose bisphosphate (FBPs) as a heterotrophic activator and it contains a cysteine in its FBP binding site [8]. Reversible modification of cysteines such as disulfide bond formation, glutathionylation, and nitrosylation may also be a means of protection from further, generally irreversible, modifications to sulfinic (–SO_2_H) or sulfonic (–SO_3_H) acids [9]. Thus, reversible cysteine modifications can influence protein activity and the relative quantification of the status of the thiol can potentially provide valuable insights into protein activity where the protein exists in a range of redox states. Redox proteomics has taken advantage of the thiol specificity of ICAT reagents not only to identify targets of ROS but also to quantify oxidative thiol modifications in individual proteins. The first applications of this technology involved exposing purified proteins to either OS or normal condition before labeling with either heavy or light ICAT reagents, respectively. This facilitated study of the activity of p21ras GTPase, a redox protein essential for cellular proliferation and differentiation which contains cysteines targeted for reversible glutathionylation and nitrosylation [10, 11]. The versatility of ICAT reagents has been further exploited in using the same technique (termed OxICAT) to determine the oxidation state of an individual protein thiol in a complex protein mixture [12].

**Figure 1:**
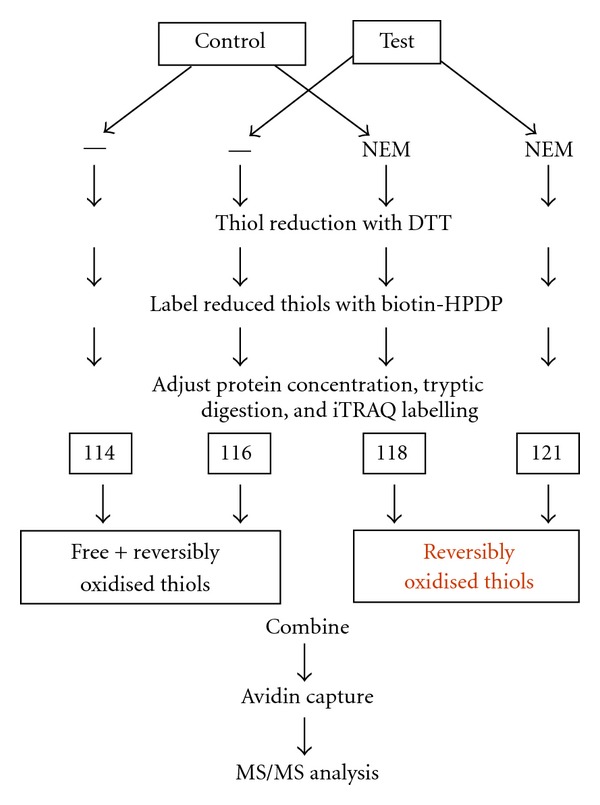
Schematic diagram for the relative quantification of the redox state of cysteine-containing peptides between two samples. Each sample (control and test) is split in two. One set has its free thiols initially blocked with the alkylating reagent NEM. Once excess NEM is removed, all samples have their reversibly oxidized thiols reduced with DTT. Free thiols in all samples are then labeled with thiol-specific biotin-HPDP, and protein concentration is measured so all samples have equivalent protein content. Proteins are tryptic digested and peptides labeled with iTRAQ reporter tags according to the scheme outlined. Labeled peptides are combined. Biotinylated cysteine-containing peptides are purified using streptavidin, and purified peptides are analysed and quantified by MS/MS.

iTRAQ has become a popular choice for researchers as it allows up to eight samples to be analyzed simultaneously. In this technique, digested peptides are labeled with amine-specific isobaric reagents to label primary amines of peptides from up to eight different biological samples [3]. We propose a novel method that exploits the accuracy and flexibility of iTRAQ together with a previous thiol selection method [13, 14] to quantify the redox state of cysteines both within and between samples in a single analysis (outlined in Figure 1). This technique allows the identification of the protein, identification of redox sensitive cysteines within the protein, and quantification of its redox state. We used yeast alcohol dehydrogenase-1 (ADH-1) as a model redox protein for proof of principle of the technique. The activity and number of free thiols in this protein decrease in a concentration-dependent manner upon exposure to H_2_O_2_. In addition, we applied the technique to a complex proteome of a Gram-negative bacterium exposed to H_2_O_2_.

## 2. Materials and Methods

All chemicals and reagents were from either Sigma or GE Healthcare unless stated and were of AnalR grade or above.

### 2.1. Alcohol Dehydrogenase

Yeast ADH-1 (100 *μ*g) in 100 mM HEPES pH 8.0 was exposed to different concentrations of H_2_O_2_ for 5 minutes and the reaction terminated by the addition of excess catalase. Enzyme activity was measured according to [15] by the formation of NADH in the first 5 minutes. Free thiol content in alcohol dehydrogenase was measured using Ellman's reagent (5,5′-dithiobis-2-nitrobenzoic acid, DTNB) at 412 nm in denaturing conditions. All activities and measurements were performed in triplicate and with *N* = 3.

### 2.2. Protein Preparation of iTRAQ

ADH-1 was prepared for analysis adapted from a method described previously [13] and outlined in Figure 1, the major difference being that Tris-HCl was replaced with HEPES due to the reactivity of iTRAQ reagents with amines. Briefly, after each treatment, the protein sample was split in two, one with a population of cysteines with free thiols blocked with NEM and the other with free thiols (without NEM). From this point on, all samples were treated identically. The protein was precipitated and washed to remove any free NEM, dissolved in 180 *μ*L denaturing buffer (8 M Urea, 4% CHAPS and 100 mM HEPES, pH 8.0) with 20 *μ*L of 200 mM DTT, and incubated for 45 min on a rotator. Protein was precipitated and washed with acetone to remove excess DTT and redissolved in denaturing buffer containing 0.5 mM biotin-HPDP (Pierce Biotechnology). Excess biotin-HPDP was removed using zebra spin trap columns (Pierce) and buffer exchanged for 100 mM HEPES, pH 8.0, using repeated cycles with microcon 3 filters. Protein concentration was measured using Bradford reagent (BioRad) [16] with BSA as a standard.

ADH-1 (10 *μ*g) from control or either 1 mM or 5 mM H_2_O_2_ exposure was tryptic digested (Promega) at a ratio of 1 : 20 trypsin : protein and incubated at 37°C for 3 hours. Peptides were labeled with iTRAQ isobaric tags (ABSciex) according to the manufactures' instructions in the following order: control (without NEM − total thiols) reporter 114, control (plus NEM − reversibly oxidized thiols) reporter 118, test 1 or 5 mM H_2_O_2_  (without NEM − total thiols) reporter 116, and 1 or 5 mM H_2_O_2_(plus NEM − reversibly oxidized thiols) reporter 121. Replicate peptides (see Supplementary information available online at doi:10.1155/2012/514847) were labeled in the same order with 113, 115, 117, and 119 iTRAQ reagents. After labeling, the four distinct isobaric-labeled peptides were combined and incubated with Streptavidin-Sepharose resin. This was prepared by washing twice in binding buffer (4 M urea, 2% CHAPS, 50 mM NaCl and 50 mM HEPES, pH 8.0), and 100 *μ*L of this slurry was incubated with peptides overnight at 4°C on a rotator. Following overnight incubation, the resin was washed once with binding buffer, twice with wash buffer A (8 M urea, 4% CHAPS, 1 M NaCl and 50 mM HEPES, pH 8.0) and three times with wash buffer B (8 M urea, 4% CHAPS, and 50 mM HEPES. In order to remove urea, the resin was washed four times with wash buffer C (5 mM HEPES/20% acetonitrile). Biotinylated peptides were eluted from the resin by adding 30 *μ*L of wash buffer C containing 20 mM DTT and incubated for 30 mins. Peptides were collected by centrifugation and stored at −70°C until analysis by MS/MS.

### 2.3. Bacterial Culture

A Gram-negative bacterial *Bacillus subtilis* strain available in our laboratory was used to assess the potential of this technique to analyze complex proteomes. Exponentially growing cells (O.D._600_ = 1–1.5) grown in standard media [17] were exposed to 1 mM H_2_O_2_ and harvested for analysis. Cell cultures were split in two for analysis, one for lysis in a buffer containing 100 mM HEPES, 8 M urea, 2 mM EDTA and 0.1% Triton and the other in the same buffer but also containing 50 mM NEM. All analyses were performed on two independent cultures. Cell lysis and protein preparation were carried out as previously described [13]. The same protocol was used for complex protein samples as with ADH-1 except 100 *μ*g of protein sample was tryptic digested and labeled with each iTRAQ reagent.

### 2.4. Sample Analysis by nLC-MALDI MS/MS

Labeled peptides were separated by reverse phase nano HPLC using the integrated autosampler Famos, switch pump, and micropump Ultimate (LC Packings). Solvent A was 10 mM Na_2_HPO_4_ in 0.1% TFA (v/v) and solvent B, 10 mM Na_2_HPO_4_ in 70% acetonitrile (ACN) and 0.1% TFA (v/v). Labeled peptides were desalted and concentrated in a reverse phase C18 PepMap column (0.3–5 mm, 5 mm, 100 Å LC Packings) for 15 min. The peptides were separated manually in a reverse phase C18 analytical column (0.075–0.1 mm, Thermo C18Aq, 5 mm, 100 Å Thermo) using a 60 min linear 6–60% gradient followed by 20 min linear increase 60–100% solvent B with a flow rate 300 *μ*L/min. Eluted fractions were collected at 12 s intervals and directly spotted onto MALDI plate OptiTOF (ABSciex) using the Suncollect system. The eluent spotted was 60 nl and mixed with 200 nL matrix *α*-cyano-4-hydroxycinnamic acid (CHCA), 7 mg/mL (w/v) in 70% ACN (v/v), and 0.1% TFA (v/v). Eluent deposition time was dependent on chromatography separation time.

nLC-MALDI fractions were analyzed using an Applied Biosystems 4800 MALDI TOF-TOF Analyzer (ABSciex) in positive ion reflector mode with a mass range of 800–4000 Da controlled by analysis programme 4000 Explorer Series v3.5 (ABSciex). A rate of 2500 laser spots per mass spectrum was used with a uniform standard. In each mass spectrum, the 20 most abundant peaks were selected for MS/MS using the ion exclusion method for ions with an S/N greater than 50, leaving out identical peaks from adjacent spots and selecting for only the highest precursor ions. Weaker precursor ions with a lower S/N ratio were acquired first to obtain a stronger signal for less abundant peptides. The peptide angiotensin was used for internal calibration of MS spectra. To obtain fragmentation MS/MS spectra, 1 kV collision energy was used. A window of 250 (total average mass width) relative to precursor ion and using CID activated collision allowed suppression of metastable ions. MS/MS spectra selected were obtained using a fixed laser shot range 1000–3000 and 50 for subspectra. The minimum criteria were set at 100 S/N in more than 7 peaks after a minimum of 1000 shots.

### 2.5. Data Analysis

The peptide data obtained by MALDI-TOF/TOF were analyzed with ProteinPilot 1.0 software using the Paragon protein database search algorithm (ABSciex). Using this software, peptide analysis data obtained with the iTRAQ system were converted into the differential analysis data for peptide matching identification and relative quantification. The parameters for the analysis were set as follows: sample type: iTRAQ 8-plex (peptide labeled); Cys alkylation: NEM and including all biological modifications; digestion: trypsin; instrument: MALDI TOF/TOF. MS/MS data were searched against all entries in the UniProt nonredundant database (517,802 sequences; 161,091,005 residues). Crude data were limited to peptide confidence (minimum 95%), the peak area of reporter ion, error of peak area of reporter ion, accession number, taxonomy, peptide sequence, assigned peptide, and the relative quantification of peptides. Rates of false positive identifications were estimated using a concatenated reversed sequence database. Only peptides with a confidence of at least 95% were used to quantify the relative abundance of each peptide determined by ProteinPilot using the peak areas of signature ions from the iTRAQ-labeled peptides.

## 3. Results

### 3.1. Alcohol Dehydrogenase

**Figure 2:**
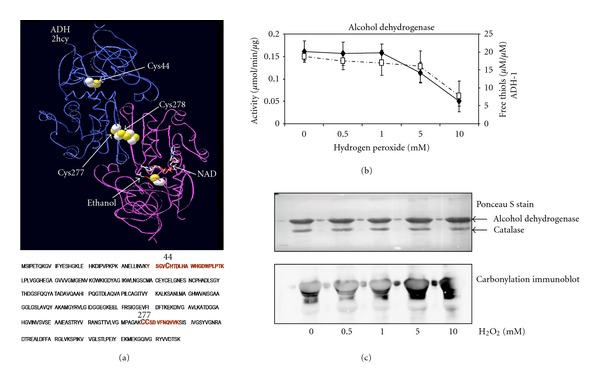
(a) ADH-1 homodimer is represented with substrate ethanol and coenzyme A at the active site. Coordinates were downloaded from the Protein Data Bank as a PDB file 2HCY and manipulated with the DeepView free software [18]. Cys^44^ and Cys^277,278^ are highlighted, and analysis of ADH-1 amino acid sequence indicates Cys-containing tryptic peptides in red that are amenable to analysis by MS/MS. Cys^44^ forms part of the catalytic centre, and Cys^277,278^ is involved in a disulfide. (b) Activity (♦−♦) and free thiols (□- -□) present in ADH-1 after exposure to increasing concentrations of H_2_O_2_. (c) Ponceau S stain and carbonylation immunoblot of ADH after H_2_O_2_ exposure; there is equivalent protein loading, but an increase in irreversible carbonylation after exposure to 5 mM H_2_O_2_ is evident.

To test the performance of the method, we used pure commercial ADH-1. Yeast ADH-1 is a tetrameric protein composed of identical 36 kDa subunits and containing two zinc ions co-coordinated to cysteine residues [15]. Of the eight cysteine residues within ADH-1, three are contained in tryptic peptides that are amenable to MS/MS analysis (Figure 2(a)). Cys^44^ contained within peptide 40–60 has been reported to coordinate to a zinc ion forming part of the catalytic centre, and oxidation plays a major role in H_2_O_2_ induced deactivation [15]. Cys^277&278^ are contained in peptide 277–287 and have been identified as forming disulfide bonds after H_2_O_2_ oxidation [15]. Exposure of ADH to H_2_O_2_ resulted in a concentration-dependent reduction in activity and free thiols. Enzyme activity decreased to about 40% after 5 minutes exposure to 5 mM H_2_O_2_ (Figure 2(b)) and free thiols as measured using Ellman's reagent also decreased and correlated with the decrease in catalytic activity (Figure 2(b)). There was also an increase in irreversible protein carbonylation at this concentration (Figure 2(c)). Once the redox behavior of the enzyme was determined, we checked whether the iTRAQ methodology could provide parallel consistent results.

### 3.2. iTRAQ Relative Quantification

**Figure 3:**
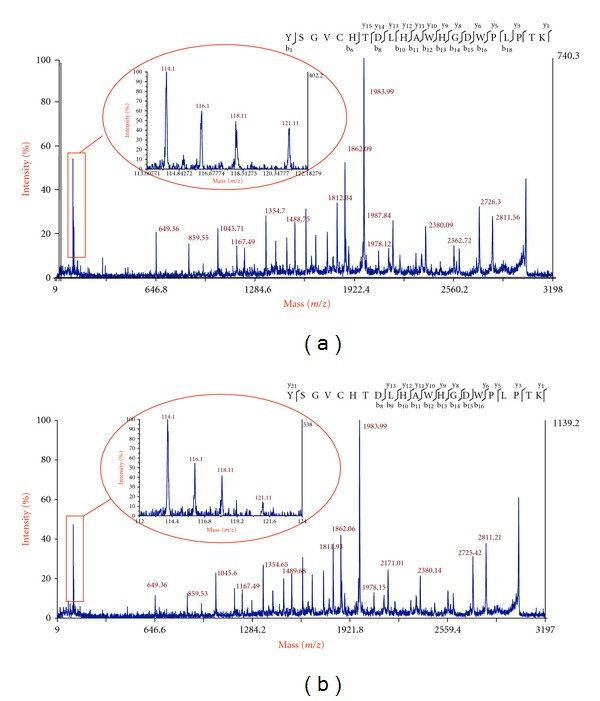
Fragmentation spectrum of peptide ^40^YSGV**C**HTDLHAWHGDWPLPTK^60^ with iTRAQ reporter ions magnified. (a) Reporter ions 114 and 118 are for controls and indicate approximately half of this Cys population is in a free thiol state. After exposure to 1 mM H_2_O_2_, there is a decrease in reporter ion 116 for total free thiols and reporter 121 indicates that it is predominantly reversibly oxidized and not present as a free thiol. (b) Reporter ions 114 and 118 are again controls and are equivalent to the control results in (a), that is, approximately half of the Cys in the peptide are in the free thiol form. After exposure to 5 mM H_2_O_2_, there is a dramatic reduction in reversibly oxidized thiols (reporter 121) indicating, at this concentration, that the Cys residue is susceptible to irreversible oxidation.

A schematic outline of our approach in applying iTRAQ reagents to relatively quantify individual cysteine-containing peptides after exposure to H_2_O_2_ is outlined in Figure 1. Samples are divided in two, one group has its free thiols blocked with NEM. Reversibly oxidized thiols are then reduced in all groups with dithiothreitol (DTT) and free thiols subsequently labeled with biotin-HPDP. After tryptic digestion, iTRAQ labeling and mixing of samples, labeled peptides are selected, analyzed, and quantified by MS/MS. Peaks are quantified relative to the control cysteine-containing peptides (labeled with 114-total thiols) which include both reduced or free thiols and reversibly oxidized thiols. The second peak (116) is the corresponding value after treatment with H_2_O_2_ (1 or 5 mM). The third peak (118) is the proportion of the peptide with reversibly oxidized thiols in controls only and the last peak (121) is the proportion of reversibly oxidized thiols after exposure to H_2_O_2_.  Table 1 lists the relative proportion of free thiols and reversibly oxidized thiols in the amenable ADH peptides. Further analysis of the results for peptide 40–60 after treatment with 5 mM H_2_O_2_ is presented in Table 2. If we take the reporter 114 from control as total detectable thiols to be 100%, then we can calculate both the proportion of that reversibly oxidized cysteine (118/114) and that in a reduced state (1 − (118/114)). Similarly, after peroxide exposure, we can calculate the proportion of the thiol remaining reversibly oxidized (121/114) and reduced (116/114)−(121/114). The remaining proportion (1 − (116/114)) is presumably over-oxidized. Inspection of the results indicates that under control conditions, approximately half of these thiols were reversibly oxidized (47%) and half were in a reduced state (53%). After exposure to 5 mM H_2_O_2_, the proportion of reversibly oxidized thiols decreased to 26%, free thiols decreased to 22%, and the overoxidized proportion was 52%. This cysteine forms part of the active site and these results correlated well with the decrease in ADH activity (~50%), loss of free thiols, and increase in carbonylation at this concentration (Figure 2). This suggests that cys^44^ is redox sensitive and subject to oxidation.  Figure 3, shows fragmentation of the precursor ion 3028 *m/z* that corresponds to peptide ^40^YSGVCHTDLHAWHGDWPLPTK^60^ in ADH-1 in control and after exposure to 1 mM (Figure 3(a)) or 5 mM H_2_O_2_ (Figure 3(b)). The reporter tags can be seen in the inset and it is clear that, after exposure to 5 mM H_2_O_2_, there is a significant decrease in iTRAQ reporter ion 121 (inset Figure 3(b)) corresponding to the relative proportion of reversibly oxidized after peroxide exposure. Exposure to 1 mM H_2_O_2_ had little effect on reversibly oxidized cysteines, coincident with lack of significant change in either enzyme activity, or in free thiols at this peroxide concentration (Figure 2(b)).

Analysis of ADH-1 peptide 277–287 is more complex due to the presence of two cysteine residues that have previously been reported to be involved in a disulfide bond [15]. The potential oxidation of either or both cysteine residues as well as thiol exchange and oxidation (especially under higher oxidative conditions) make the relative quantification complex for this technique. In-depth analysis of this peptide after differential alkylation of cysteines by selective MS/MS ion monitoring (SMIM) [19] indicated that the two cysteines can exist alternatively in both reduced and reversibly oxidized forms. Application of SMIM indicated the peptide exists in at least twelve distinct oxidation states and even with both cysteines in a –SO_3_H form after 5 mM H_2_O_2_ (Supplementary information Figures 1 and 2). This is further supported by our results after application of iTRAQ in which we have seen both alternative cysteine residues irreversibly oxidized to –SO_2_H forms and a consistent relative increase in the peptide signal after exposure to 5 mM H_2_O_2_. Taken together with the fact that at least one of the thiols needs to be either in a reduced state or reversibly oxidized to be able to capture the cysteine-containing peptide, analysis of the redox state of individual cysteines in such peptides is complex.

Application of this technique to the redox proteome of *B. subtilis* resulted in identification and relative quantification of the redox status of 23 cysteine-containing peptides from 18 known redox-sensitive proteins (Supplementary Table 1). A number of these proteins known to be sensitive to redox changes and have been well characterized, for example, thiol peroxidase, elongation factors, and ribosomal proteins. Application of the same criteria used in Table 2 for a selection of these cysteine-containing peptides, is presented in Table 3. In general, results are as would be expected with a large number of proteins having a decreased value for total detectable thiols (116 : 114) ratio after exposure to 1 mM H_2_O_2_. We also have an estimation of the proportion of the total thiols that are reversibly oxidized in both controls (118 : 114 ratio) and after peroxide exposure (121 : 116 ratio). The advantage of this technique can clearly be seen when we examine the peptides for elongation factor G (Q8CQ82) protein 5 with two cysteine-containing peptides detected. Relative quantification of the cysteines within the two peptides indicates that under control conditions, the majority of the thiols are reversibly oxidized (85% and 95%, resp.) In the first peptide ^595^CNPVILEPISK^605^ the proportion of thiols reversibly oxidized did not change dramatically after exposure 75% (121 : 114) and approximately 25% of the thiols were over-oxidized. However, quantification of the second peptide ^381^DTTTGDTL**C**DEK^392^ indicates that after exposure, the proportion of reversibly oxidized thiols decreased from 95% (118 : 114) to 30% (121 : 114) while the proportion irreversibly oxidized (–SO_2_H or –SO_3_H) increased to 75%. Elongation factor G is redox sensitive and known to be inactivated by sulfhydryl reagents in other species [20, 21]. Yet this technique allowed us to identify the redox sensitive cysteine within the protein, which would not be detected by relative quantification alone. Elongation factor Tu is also known to be redox sensitive, and indeed both elongation factors G and Tu have previously been purified using covalent chromatography with thiol sepharose beads [22, 23] indicating that they possess free thiols and are redox dependent. It is known that EfTu cys^81^ and cys^137^ are associated with aminoacyl-tRNA and guanosine nucleotide binding, respectively, in *Escherichia coli* [24] equivalent to the peptides containing cys^82^ and cys^138^ detected here. Interestingly, cys^81^ has been reported as the site for nucleotide binding in *E. coli* and the equivalent cys^82^ increases in relative abundance in both control and treated samples even after initial alkylation with NEM, which is probably due to over-oxidation of sensitive thiol groups during the relatively harsh conditions used for cell lysis, resulting in an under estimation for the reference “total detectable thiols” and hence an artificially higher value for the proportion of thiols reversibly oxidized.

## 4. Discussion

Cysteines are one of the most rarely used amino acids in proteins [25]. Therefore, when conserved, they usually play critical roles in structure, function, or regulation of the protein. The average pK_*a*_ value of cysteines has been calculated as 6.8 ± 2.7, indicating that at physiological pH, they may exist in both charged thiolate form and uncharged form depending on a number of factors [26, 27]. The location and sequence of surrounding amino acids strongly influence the pK_*a*_ and hence, reactivity of a particular cysteine residue. In unstressed mammalian cells, it has been demonstrated that proteins disulfides (PSSP) account for 6% and 9.5% of protein sulfhydryls in HEK and HeLa cells, respectively. After treatment with the thiol-specific oxidant diamide, this increased to 24% and 25%. The steady state level of glutathione-protein mixed disulfides (PSSG) was less than 1% but this increased to 15% after prooxidant treatment [28]. Protein thiols therefore represent an important and significant redox buffer within the cell so application of a relative quantification method is now especially timely. iTRAQ is a flexible and multiplexed quantitative method and we had successfully developed a high throughput method for oxidized cysteine selection. A combination of both techniques could in principle be appropriate for quantitatively analyze the redox proteome. Here we demonstrate that the combined approach is feasible and provides useful information, despite some limitations.

Key goals in identifying redox-regulated proteins involve determining which proteins are involved, which cysteines within those proteins are redox sensitive, and identifying thiol modifications within particular cysteines [29]. Although the technique described herein cannot distinguish the type of reversible modifications of cysteines, it does allow for quantification of the proportion of the cysteine that is reversibly modified (and also free thiols) in both control and test conditions. Each cysteine-containing peptide is monitored independently so it is applicable to proteins that contain various cysteines reacting at different rates or which are involved in different protein functions. The relative merits and drawbacks regarding precision and accuracy of iTRAQ reagents have been extensively studied elsewhere [30, 31]. This paper aims to present the results of a novel application of these reagents in redox proteomics. Our results indicate that, when this technique is applied to study the redox state of purified proteins (in this case ADH-1), quantification of the catalytic cys^44^ with iTRAQ correlates with observed decrease in enzyme activity and loss of free thiols. When applied to a complex proteome, it can identify and relatively quantify the redox state of amenable cysteines within abundant proteins. Abundant proteins are both predominantly identified and quantified because iTRAQ labeling is optimized for a maximum of 100 *μ*g protein and we are dealing with a small percentage of amenable peptides form the total proteome. Disulfides in proteins have been classified as forming subproteomes, redox responsive, or the more resistant structural disulfides [32]. One of the advantages of the technique employed in this analysis is that redox-responsive cysteines can be distinguished from structural cysteines by change in relative abundance not only after initial blocking but also after exposure to OS. For instance, elongation factor G has two very distinct cysteine peptides in terms of their sensitivity to OS; cys^389^ is more sensitive to oxidation by OS than cys^595^. This is also an important aspect when proteins have an altered function dependent on their redox state. For instance, it is known that the peroxiredoxins may act as peroxidases, redox sensors, or chaperones depending on oligomerization, which is, in turn, dependent on the redox state [33].

Our approach also provides meaningful information regarding both the sensitivity and oxidation states of individual cysteine residues and may provide clues to regulation and catalytic centres when there is no structural information available for a given protein. When applying this technique to quantification of sensitive cysteines in complex mixtures, care must be taken to minimize oxidation during cell lysis. One shortcoming of the technique is that, when there are two or more cysteine residues within a peptide it cannot distinguish the cysteine involved and so quantification of the redox state is not possible. This was demonstrated with a two-cysteine-containing peptide from ADH-1 that existed in up to twelve distinct states after differential oxidation. Nevertheless, this technique provides both an informative and powerful tool in the study of redox proteomics with all the advantages of the iTRAQ reagents and protocols regarding precision, accuracy, multiplexing, and availability in conventional Proteomics facilities.

## Supplementary Material

Supplementary Figure 1: Identification of peptide CCSDVFNQVVK doubly labelled with NEM and IAM. ADH was first treated with NEM to alkylate free thiols and then reduced with DTT to freed disulfides and blocked with IAM. MS/MS traces from selected peptide fragments are shown in control (A); in 1 mM (B) and 5 mM (C) H_2_O_2_ treated samples. (1) Trace of fragment y6^+^. (2) Trace of fragment y9^+^. (3) Trace of fragment y10^+^(IAM-CSDVFNQVVK). (4) Trace of fragment b1^+^ (NEM-C). This data demonstrate that both successive cysteines can coexist in different redox states: one reduced and the other reversibly oxidised.
Supplementary Figure 2: Chromatograms corresponding to the base peak signals of most intense precursor ions of (A) control, (B) 1 mM H_2_O_2_ treatment, (C) 5 mM H_2_O_2_ treatment, and MS/MS trace from doubly sulfonic acid CCSDVFNQVVK fragments in control (D), 1 mM H_2_O_2_ treatment (E) and 5 mM H_2_O_2_ treatment (F). These results show how the peptide population of ADH changes upon treatment with H_2_O_2_ and how the doubly sulfonic acid modified peptide is only detected in the 5 mM H_2_O_2_ treated sample.
Supplementary Table 1: List of proteins and the Cys-containing peptides used for identification from the gram negative *B. subtilis*. Quantification is relative to control total and reversibly-oxidized thiols (total detectable thiols), taken as 1.0. The 116:114 is the total detectable thiols after exposure to 1 mM H_2_O_2_ compared to control. 118:114 and 121:114 are the ratios of reversibly-oxidized thiols in control and after H_2_O_2_ exposure, respectively, in comparison to total free thiols and reversibly-oxidized thiols in controls. Blast searches confirmed all peptides are found in *B. subtilis*.

## Figures and Tables

**Table 1:** Relative quantification of the redox state of Cys-containing tryptic peptides from yeast ADH-1 after exposure to either 1 or 5 mM H_2_O_2_. The ratio of free and reversibly oxidized thiols are compared to control levels (taken as 1.0^∗^). 116 : 114 are the relative amounts of total thiols after H_2_O_2_ exposure. Shaded boxes are the relative amounts of reversible oxidized thiols only, referred to total thiols in control; thus, 118 : 114 and 121 : 114 are the relative amounts of reversibly oxidized thiols in controls and after exposure, respectively.

Amenable Cys tryptic peptides from yeast ADH-1	Free + reversibly oxidized thiols^∗^			Reversibly oxidized thiols		
	Control (114 : 114)	1 mM H_2_O_2_ (116 : 114)	5 mM H_2_O_2_ (116 : 114)	Control (118 : 114)	1 mM H_2_O_2_ (121 : 114)	5 mM H_2_O_2_ (121 : 114)
^ 40^YSGV**C**HTDLHAWHGDWPLPTK^60^	1.00	0.341 ± 0.029	0.478 ± 0.143	0.467 ± 0.18	0.548 ± 0.221	0.258 ± 0.195
^ 277^ **CC**SDVFNQVVK^287^	1.00	0.288 ± 0.021	2.293 ± 1.041	0.397 ± 0.15	0.549 ± 0.139	0.346 ± 0.293

**Table 2:** Relative quantification of the redox status of cys^44^ in the ADH-1 peptide (40–60) in controls and after exposure to 5 mM H_2_O_2_. There is a decrease in both reversibly oxidized (47% to 26%) and reduced thiols (53% to 22%) and an increase in over oxidized thiols (52%) at this peroxide concentration.

Protein/peptide	Total detectable thiols (%)	Sample	Reversibly oxidized thiols (%)	Free thiols (%)	Overoxidized thiols (%)
Example	100	Control	(118/114)	1 − (118/114)	Not detectable
		Test	(121/114)	(116/114) − (121/114)	1 − (116/114)
ADH-1 (40–60)	100	Control	47	53	ND
		Test (5 mM H_2_O_2_)	26	22	52

**Table 3:** A selection of peptides identified from the Gram-negative bacteria, *B. subtilis* with relative quantification of the redox state of identified cysteine peptides. Total detectable thiols refer to both reversibly oxidized and reduced thiols and quantification is relative to control values. As overoxidized thiols are not amenable to selection they are not detected in controls (N.D.).

Protein (accession number)	Cys tryptic peptide	Total detectable thiols (%)	Sample	Reversibly oxidized thiols (%)	Free thiols (%)	Overoxidized thiols (%)
Triose phosphate isomerase (Q65ENO)	^ 85^DLGVEY**C**VIGHSER^98^	100	Control	41	59	N.D.
			Test	35	32	33
	^ 179^SSTSEDANEM**C**AHVR^193^	100	Control	51	49	N.D.
			Test	52	32	17
Elongation factor Ts (Q65JJ8)	^ 15^TGAGMMD**C**K^23^	100	Control	53	47	N.D.
			Test	52	8	40
	^ 234^YFEEI**C**LLDQAFVK^247^	100	Control	66	34	N.D.
			Test	44	4	52
Elongation factor G (Q65PB0)	^ 595^ **C**NPVILEPISK^605^	100	Control	85	15	N.D.
			Test	75	−10	25
	^ 381^DTTTGDTL**C**DEK^392^	100	Control	95	5	N.D.
			Test	30	−6	75
Elongation factor Tu (Q5P334)	^ 138^ **C**DMVDDEELLELVEMEVR^155^	100	Control	117	(−17)	N.D.
			Test	91	−1	8
	^ 76^HYAHVD**C**PGHADYVK^90^	100	Control	173	(−73)	N.D.
			Test	150	(−41)	−50
Adenylate kinase (P35140)	^ 75^ND**C**DGGFLLDGFPR^88^	100	Control	94	4	N.D.
			Test	68	1	31
Purine nucleoside phosphorylase (Q65IE9)	^ 27^YIADTYLENVE**C**YNEVR^43^	100	Control	91	9	N.D.
			Test	50	9	41
Transition state regulatory protein AbrB (P08874)	^ 50^YKPNMT**C**QVTGEVSDDNLK^68^	100	Control	39	61	N.D.
			Test	46	23	31
